# The NAC transcription factor ANAC046 is a positive regulator of chlorophyll degradation and senescence in Arabidopsis leaves

**DOI:** 10.1038/srep23609

**Published:** 2016-03-29

**Authors:** Chihiro Oda-Yamamizo, Nobutaka Mitsuda, Shingo Sakamoto, Daisuke Ogawa, Masaru Ohme-Takagi, Akemi Ohmiya

**Affiliations:** 1 National Agriculture and Food Research Organization (NARO), Institute of Floricultural Science, Tsukuba, Ibaraki 305-8519, Japan; 2 Research Fellow of Japanese Society for the Promotion of Science (JSPS), Tokyo 102-0083, Japan; 3 Bioproduction Research Institute, National Institute of Advanced Industrial Science and Technology (AIST), Tsukuba, Ibaraki 305-8566, Japan; 4 Horticultural Experiment Center, Wakayama Prefectural Agricultural Research Station, Gobo, Wakayama 644-0024, Japan; 5 Institute for Environmental Science and Technology (IEST), Saitama University, Saitama, Saitama 338-8570, Japan

## Abstract

Chlorophyll (Chl) degradation occurs during leaf senescence, embryo degreening, bud breaking, and fruit ripening. The Chl catabolic pathway has been intensively studied and nearly all the enzymes involved are identified and characterized; however, the molecular regulatory mechanisms of this pathway are largely unknown. In this study, we performed yeast one-hybrid screening using a transcription factor cDNA library to search for factors controlling the expression of Chl catabolic genes. We identified ANAC046 as a common regulator that directly binds to the promoter regions of *NON-YELLOW COLORING1*, *STAY-GREEN1* (*SGR1*), *SGR2*, and *PHEOPHORBIDE a OXYGENASE*. Transgenic plants overexpressing *ANAC046* exhibited an early-senescence phenotype and a lower Chl content in comparison with the wild-type plants, whereas loss-of-function mutants exhibited a delayed-senescence phenotype and a higher Chl content. Microarray analysis of *ANAC046* transgenic plants showed that not only Chl catabolic genes but also senescence-associated genes were positively regulated by ANAC046. We conclude that ANAC046 is a positive regulator of Arabidopsis leaf senescence and exerts its effect by controlling the expression of Chl catabolic genes and senescence-associated genes.

Loss of green color, the most visually apparent phenomenon during leaf senescence, is caused by chlorophyll (Chl) degradation. This process facilitates redistribution of nutrients from senescent leaves to reproductive organs and increases reproductive success^1^. In addition, loss of Chl in flowers and fruits is an important trait that enables them easily visible among leaves to attract pollinators and seed dispersers.

During past three decades, identification of Chl catabolite structures^2^ and analysis of stay-green mutants, in which Chl degradation is impaired^3^,^4^, have facilitated the elucidation of the Chl catabolic pathway. To date, nearly all enzymes involved in Chl degradation have been identified and characterized. In higher plants, reduction of Chl *b* to Chl *a* is the initial step of Chl breakdown (Fig. 1). Chl *b* reductase, encoded by *NON-YELLOW COLORING 1* (*NYC1*) and *NYC1-like* (*NOL*), catalyzes the conversion of Chl *b* to 7-hydroxymethyl Chl *a*^5^,^6^, which is then converted into Chl *a* by 7-hydroxymethyl-chlorophyll *a* reductase (HCAR)^7^. The central Mg atom of Chl *a* is removed by a metal-chelating substance (MCS), the molecular nature of which has not been identified, to form pheophytin *a*. Pheophytinase (PPH) then catalyzes the removal of the phytol tail of pheophytin *a* to form pheophorbide *a*^8^,^9^. Subsequently, the porphyrin macrocycle ring of pheophorbide *a* is opened by pheophorbide *a* oxygenase (PaO) to produce red chlorophyll catabolite (RCC)^10^,^11^. RCC is converted to nonfluorescent chlorophyll catabolite (NCC) or nonfluorescent dioxobilin-type chlorophyll catabolite (NDCC), end-products of Chl breakdown, via *primary* fluorescent Chl catabolite (*p*FCC) by the actions of RCC reductase (RCCR), MES16, and CYP89A9^12^,^13^,^14^. Chl breakdown also requires a protein termed STAY GREEN (SGR). *Arabidopsis* has three SGR homologs, SGR1, SGR2, and SGR-like^15^. SGR1 and SGR-like positively regulate Chl degradation in senescing and pre-senescing leaves, respectively, whereas SGR2 negatively regulates Chl degradation in senescing leaves^16^,^17^. Many Chl catabolic genes, including *NYC1*, *PPH*, *PaO*, *MES16*, and *SGR1* are coordinately expressed and their transcript levels increase in senescing leaves^4^.

Senescence is accompanied by transcriptional reprogramming of a large number of genes. In senescent leaves of Arabidopsis, more than 100 transcription factors (TFs) from various families, including NAC (no apical meristem [NAM], ATAF1/2, and cup-shaped cotyledon [CUC2]), WRKY, zinc finger proteins, and AP2/EREBP are upregulated^18^. In particular, plant-specific NAC superfamily constitutes a large portion of senescence-regulated genes in Arabidopsis^18^,^19^,^20^. Several NAC TFs, including AtNAP/ANAC029^21^, ORESARA1/ANAC092^20^,^21^,^22^,^23^, ORE1 SISTER1/ANAC059 (ORS1)^24^, and AtNAC016^25^, positively regulate leaf senescence. Their downstream target genes, including senescence-associated genes (SAGs), have been identified^20^. Only limited information is available on whether the senescence-related NAC TFs are involved in the control of Chl catabolic gene expression. Recently, Qiu *et al*.^26^ reported that ORE1 directly binds to the promoter regions of *SGR1*, *NYC1*, and *PaO*.

Towards an understanding the mechanism of Chl degradation, we performed comprehensive screening of TFs that bind to the promoter regions of Chl catabolic genes using an improved yeast one-hybrid (Y1H) screening system with an Arabidopsis TF cDNA library^27^. Because the TF cDNA library is composed of 10% or less number of all Arabidopsis genes, the rate of false positives is much lower than in traditional screening. Another advantage of this approach is that we can directly employ the promoter region as bait. Here, we report identification of ANAC046, a previously uncharacterized member of the NAC superfamily, as a common regulator of the expression of *NYC1*, *SGR1*, *SGR2*, and *PaO*. ANAC046 belongs to the same subgroup as ORE1 and ORS1. Our analysis of mutant and transgenic *ANAC046* plants showed that ANAC046, ORE1, and ORS1 share common functions but also have distinct roles in leaf senescence.

## Results

### Identification of transcription factors that bind to the promoter regions of chlorophyll catabolic genes

To identify the TFs that directly control Chl degradation, we first constructed yeast strains carrying the *HIS3* reporter gene driven by the promoter regions of the following Chl catabolic genes: *NYC1*, *NOL*, *HCAR*, *PPH*, *PaO*, *RCCR*, *CYP89A9*, *MES16*, *SGR1*, and *SGR2*. Then, we examined binding of ca. 1300 TFs (ca. 70% of all Arabidopsis TFs) to these regions. After the initial screening with 343 mini-pools, we performed a second screening and identified 93 interactions between the 10 promoters and 54 individual TFs (Fig. S1). Among them, we were interested in the particular group of NAC TFs because three genes namely ANAC046, ANAC087, and ANAC100 were detected in experiments employing the promoters of *NYC1*, *SGR1*, *SGR2*, and *PaO* (Fig. S1). Phylogenetic analysis clustered these three proteins into the same clade as ANAC079, ORE1, and ORS1 in the NAM subgroup of group I of the NAC superfamily (Fig. S2)^28^. *ANAC079*, *ANAC100*, and *ORE1* have miR164-complementary sites with ≤3 mismatches to miR164 and are likely to be target genes for this miRNA (Fig. S3).

**Figure 2:**
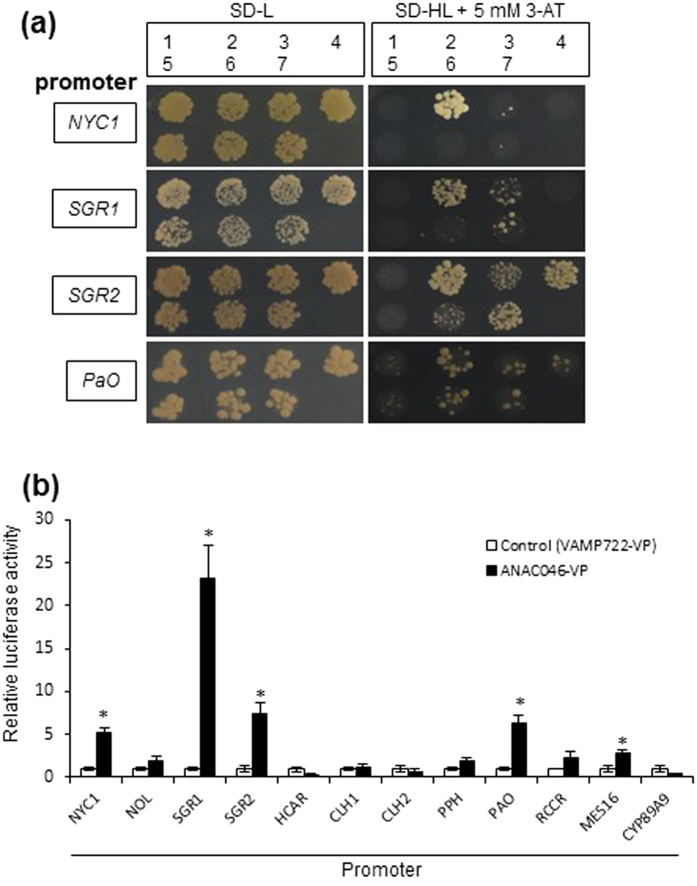
ANAC046 activates the promoters of chlorophyll catabolic genes. **(a)** Activation of NAC transcription factors against promoters of chlorophyll catabolic genes was tested in Y1H experiment. The following promoters were tested: *NYC1*, *SGR1*, *SGR2*, and *PaO*. The transcription factors tested were as follows: 1, none (empty vector pGAD424); 2, ANAC046; 3, ANAC087; 4, ORE1; 5, ORS1; 6, ANAC079; and 7, ANAC100. Growth of yeast transformants carrying the *HIS3* reporter gene under the control of each promoter region was examined in the medium (SD) lacking histidine (H) and leucine (L) but containing 3-AT. **(b)** Transactivation of the Chl catabolic gene promoter–firefly luciferase fusion genes by the ANAC046 protein in Arabidopsis leaf protoplasts. The *LUC* reporter gene under the control of the Chl catabolic gene promoters was co-transfected with an effector plasmid expressing ANAC046-VP16 or VAMP722-VP16 (negative control). To normalize transfection efficiency, the CaMV 35S promoter–Renilla luciferase plasmid was cotransfected in each experiment and firefly luciferase activity was normalized to that of Renilla luciferase. Gene designations are as in Fig. 1. Bars indicate standard error (4 biological replicates). Asterisks indicate significant differences (*P* < 0.01) between VAMP722-VP and ANAC046-VP according to Student’s *t*-test.

Next, we examined the binding of six TFs to four promoters in a separate Y1H experiment (Fig. 2a). ANAC046 bound to all the promoters tested. For ANAC087, the TF most similar to ANAC046 (Fig. S2), the affinity to all of the promoters was low. ORE1 bound to the *SGR2* and *PaO* promoters, and ORS1 did not bind to any of the promoters tested. Therefore, we chose ANAC046 for further analysis.

To examine the interactions between ANAC046 and promoters of the 12 chlorophyll catabolic genes, we employed transient effector–reporter analysis in leaf protoplasts. As shown in Fig. 2b, the VP16-fused form of ANAC046 clearly activated the reporter driven by the promoters of *NYC1*, *SGR1*, *SGR2*, and *PaO*, suggesting *in planta* binding of ANAC046 to these promoters. Thus, the results obtained in leaf protoplasts were consistent with those obtained in yeast. ANAC046 also activated *MES16* promoter *in planta* with less extent, although this activation was not observed in yeast.

### Phenotypic Analysis of *ANAC046* Transgenic Plants

To elucidate the function of ANAC046 in Chl degradation, we produced transgenic Arabidopsis plants constitutively overexpressing *ANAC046* (*35S:ANAC046*; OX) and plants expressing a chimeric repressor containing the SRDX domain at the C-terminus (*35S:ANAC046-SRDX*; SRDX). The SRDX plant is expected to show loss-of-function phenotype of *ANAC046* and its functionally redundant transcription factors as a result of dominant suppression of its target genes^29^. We also obtained a putative knockout mutant, SM_3_27647 (KO), which has a transposon insertion in the *ANAC046* coding region.

**Figure 3:**
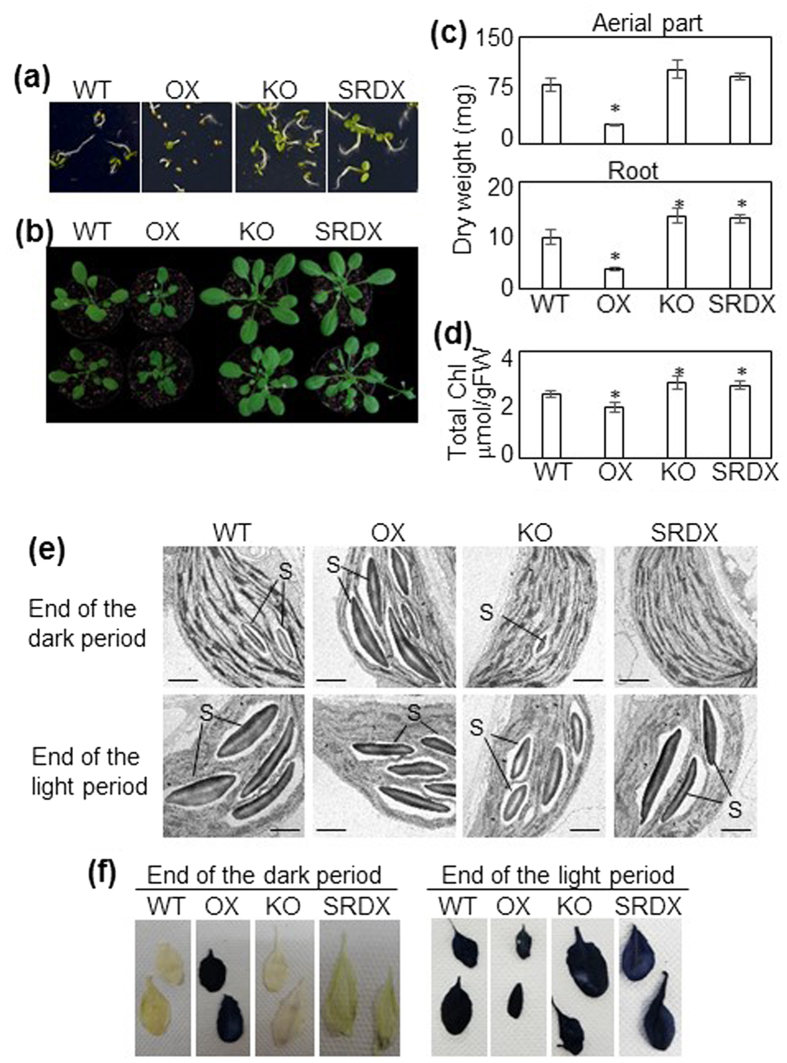
Phenotypes of *ANAC046* transgenic plants. **(a)** Seedlings grown on MS-agar 4 days after sowing (DAS). **(b)** Plants grown in soil at 40 DAS. **(c)** Dry weight of aerial parts and roots of 40-DAS plants. Bars indicate standard error (10 biological replicates). **(d)** Chlorophyll content in the 8–9th leaves of 40-DAS plants. Bars indicate standard error (4 biological replicates). The presence of starch in rosette leaves of 40-DAS plants detected by **(e)** transmission electron microscopic imaging of chloroplasts and **(f)** iodine staining. S, starch granules. Scale bars = 1 μm. Asterisks indicate significant differences between WT and OX, KO, or SRDX according to Student’s *t*-test (*P < 0.05).

The OX plants germinated on MS-agar slightly later than WT plants (Fig. 3a). At 40 days after sowing (DAS), the OX plants had wavy rosette leaves and were remarkably smaller than WT plants (Fig. 3b). The KO and SRDX plants were slightly larger than WT plants during the course of development. The dry weight of aerial parts and roots was significantly lower in OX plants and that of roots was significantly higher in KO and SRDX plants than in WT plants (Fig. 3c). Chl content in the 8–9th leaf at 40 DAS was slightly higher in the KO and SRDX plants and significantly lower in OX plants than in the WT plants (Fig. 3d).

To compare the ultrastructure of chloroplasts, the leaves of WT, OX, KO, and SRDX plants at 40 DAS were analyzed under a transmission electron microscope (TEM). At the end of the light period, chloroplast structure was similar in all plants tested: all mesophyll chloroplasts contained several large starch granules (Fig. 3e), which were produced by photosynthesis during the day. At the end of the dark period, only small starch granules were visible in the chloroplasts of the WT, KO, and SRDX plants, whereas chloroplasts of the OX plants still contained several large starch granules (Fig. 3e). In line with these observations, iodine staining showed that the leaves of OX plants, but not those of the WT, KO, or SRDX plants, contained a high level of starch at the end of the dark period, whereas the starch level was high in the leaves of all plants at the end of the light period (Fig. 3f).

### Involvement of ANAC046 in leaf senescence

**Figure 4:**
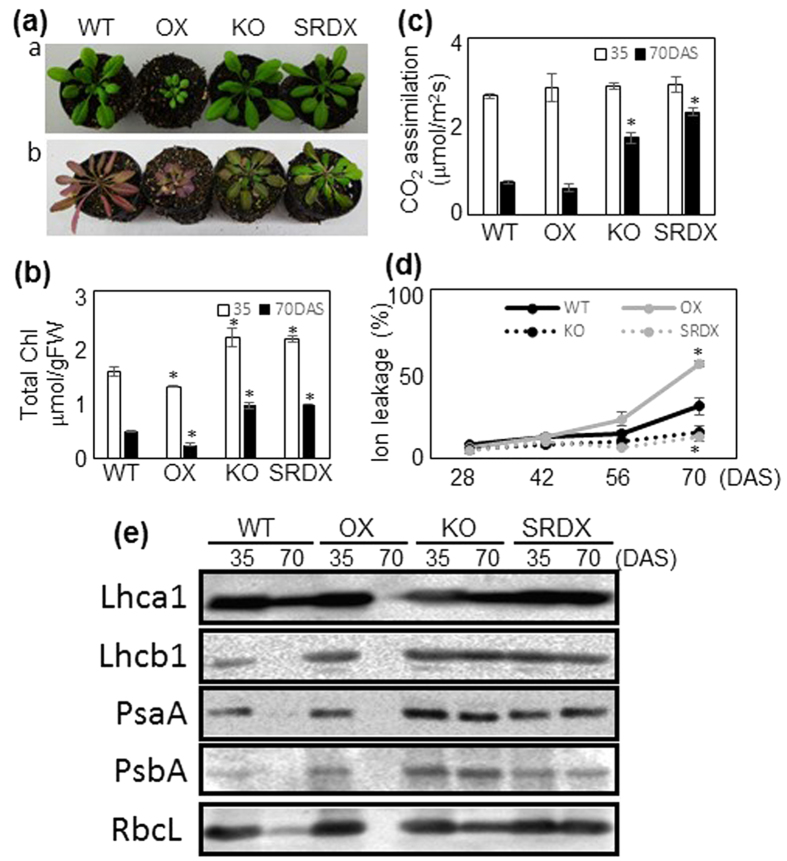
Age-dependent senescence in WT and *ANAC046* transgenic plants. **(a)** Plants at 35 DAS (a) and 70 DAS (b). **(b)** Chlorophyll content and **(c)** CO_2_ assimilation rate of 35- and 70-DAS plants. Bars indicate standard error (4 biological replicates). Asterisks indicate significant differences between WT and OX, KO, or SRDX plants at each sampling time according to Student’s *t*-test (*P < 0.05). **(d)** Ion leakage rate of 28-, 42-, 56-, and 70-DAS plants. **(e)** Western blotting of photosynthetic proteins. Protein extract equivalent to 1.0 mg of leaf tissue per lane was subjected to SDS-PAGE.

To determine the involvement of ANAC046 in age-dependent senescence, we compared the plant phenotypes and Chl content in the leaves of OX, KO, SRDX, and WT plants at 35 and 70 DAS. Leaf yellowing was accelerated in the OX plants and delayed in the KO and SRDX plants in comparison with the WT plants (Fig. 4a). Leaf Chl content was significantly higher in the KO and SRDX plants than in the WT plants at both 35 and 70 DAS (Fig. 4b). At 70 DAS, Chl content in the leaves of the OX plants was approximately half that of the WT plants. CO_2_ assimilation rate was not significantly different among WT, OX, KO, and SRDX plants at 35 DAS, but was significantly higher in the KO and SRDX plants and slightly (but not significantly) lower in the OX plants than in the WT plants at 70 DAS (Fig. 4c). Ion leakage rate (a senescence indicator) was similar in all plants until 56 DAS (Fig. 4d). At 70 DAS, it was significantly higher in the OX plants but significantly lower in the KO and SRDX plants than in the WT plants.

The levels of photosynthetic proteins associated with PSI and PSII were assessed by western blotting with antibodies against the core proteins (PsaA and PsbA, respectively) and the antenna proteins of the light-harvesting complexes (Lhca1 and Lhcb1). The levels of the large subunit of Rubisco (RbcL), which is involved in carbon fixation, were also examined. At 35 DAS, we did not detect remarkable differences in the levels of the photosynthetic proteins among plants tested (Fig. 4e). At 70 DAS, much higher levels of all proteins were detected in the KO and SRDX plants than in the WT plants, whereas all the proteins tested were below the detection limit in the OX plants.

**Figure 5:**
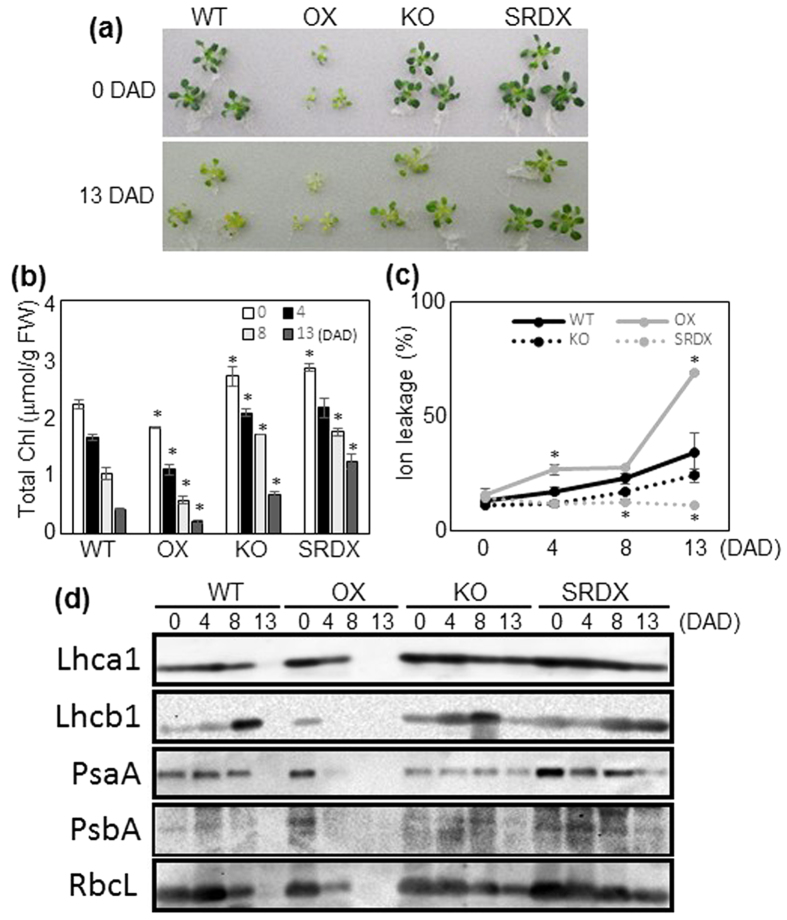
Dark-induced senescence in WT and *ANAC046* transgenic plants. **(a)** Plants at 0 DAD (35 DAS) and 13 DAD. **(b)** Chl content and **(c)** ion leakage rate of 0-, 4-, 8-, and 13-DAD plants. Bars indicate the standard error (4 biological replicates). Asterisks indicate significant differences between WT and OX, KO, or SRDX plants at each sampling time according to Student’s *t*-test (*P < 0.05). **(d)** Western blotting of photosynthetic proteins. Protein extract equivalent to 1.0 mg of leaf tissue was subjected to SDS-PAGE.

We also analyzed the effect of ANAC046 on dark-induced senescence by transferring the plants at 35 DAS to continuous darkness. Leaves of the OX plants grown on MS-agar were pale green and somewhat smaller than WT plants at 35 DAS (Fig. 5a). Chl content decreased faster in the OX plants, but slower in the KO and SRDX plants, than in the WT plants (Fig. 5b). The SRDX plants retained a high level of Chl even at 13 days after dark treatment (DAD). Ion leakage rate was significantly higher in the OX plants at 4 and 13 DAD and significantly lower in SRDX plants at 8 and 13 DAD than in the WT plants (Fig. 5c). The levels of all the photosynthetic proteins tested drastically decreased at 4 DAD in the OX plants but remained constant in the WT, KO, and SRDX plants (Fig. 5d). They were below the detection limit at 8 DAD in the OX plants. In contrast, the KO and SRDX plants contained substantial amounts of photosynthetic proteins even at 13 DAD, when these proteins became undetectable in WT plants.

**Figure 6:**
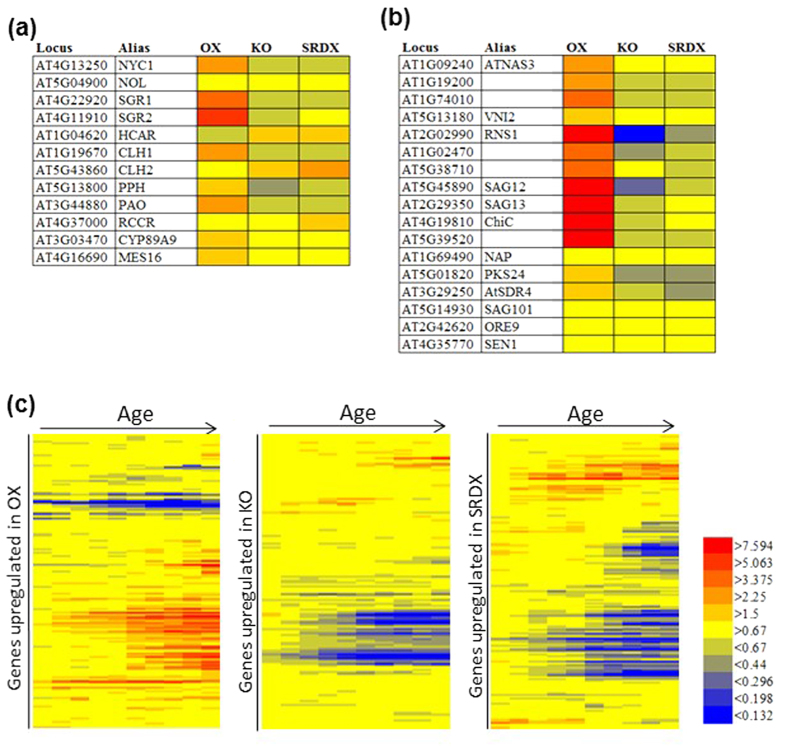
Microarray analysis of *ANAC046* transgenic plants. Heat maps of fold changes in the expression of **(a)** Chl catabolic genes, **(b)** other senescence-related genes, and **(c)** age-dependent changes in the expression levels of genes upregulated in the OX, KO, and SRDX plants. Gene expression data along plant age (**c**) were obtained from a public database^30^. Fold change of expression in afternoon of 21, 23, 25, 27, 29, 31, 33, 35, 37, and 39 days after sowing (DAS) over one in afternoon of 19DAS is shown by color from left to right.

To obtain a comprehensive view of transcriptomic changes in transgenic plants, we performed microarray experiments (Fig. 6). We used RNAs from three independent lines of the OX and SRDX plants for the analysis. The level of *ANAC046* expression in these transgenic plants was more than 17-fold higher than that of WT (Fig. S4). We also confirmed that *ANAC046* expression in the KO plants was extremely low. We found that SRDX and KO plants, which had similar phenotypes, also had similar transcriptomes (r = 0.71; Fig. S5). The expression of Chl catabolic genes, including *NYC1*, *CLH1*, *PPH*, *PaO*, *SGR1*, and *SGR2* was markedly higher in the OX plants and lower in the KO and SRDX plants than in the WT plants (Fig. 6a). Likewise, the expression of marker genes for leaf senescence, including *SAG12* and *SAG13*, was also higher in the OX plants and lower in the KO and SRDX plants than in WT plants (Fig. 6b). We also examined age-dependent changes in the expression of genes upregulated in each of the three transgenic lines by using publicly available microarray data of wild type (Fig. 6c)^30^. The expression of most genes upregulated in the OX plants increased with age, whereas the expression of most genes upregulated in the KO and SRDX plants decreased with age (Fig. 6c). These data clearly support accelerated senescence of OX plants and delayed senescence of KO and SRDX plants.

## Discussion

The Chl degradation pathway consists of six chloroplast-located enzymes, two cytoplasm-located enzymes, a metal-chelating substance, and the key regulator SGR, and produces nonfluorescent chlorophyll catabolites^3^,^4^,^31^. In Arabidopsis leaves, the expression of Chl catabolic genes is highly coordinated during senescence^4^, suggesting the existence of TFs that bind to the promoter regions and coordinate the expression of a number of Chl catabolic genes. In this study, we found that several NAC TFs, including ANAC046, ANAC087, and ANAC100, directly bind to the promoter regions of *NYC1*, *SGR1*, *SGR2*, and *PaO*. It is worthy of note that the expression of these Chl catabolic enzymes is well correlated with that of these three NAC TFs, with both groups showing a drastic increase during leaf senescence^4^. Our analyses of mutant and transgenic ANAC046 plants indicate that ANAC046 is a positive regulator of leaf senescence.

ANAC046 belongs to the same subgroup as ORE1 and ORS1 (Fig. S2), TFs associated with leaf senescence^20^,^22^,^23^,^24^. Recently, Qiu *et al*.^26^ showed that ORE1 directly promote the expression of a similar set of Chl catabolic genes. Our microarray analysis of *ANAC046* mutant plants showed that many genes inducible by ORE1, ORS1, or both were also upregulated in *ANAC046* OX plants and downregulated in *ANAC046* SRDX and KO plants (Fig. S6 and Table S1) suggesting that these TFs have overlapping functions during age-dependent senescence. However, there may be no or only limited functional redundancy between ORE1 and ANAC046 in terms of age-dependent senescence because distinct delayed senescence phenotypes were observed in an single mutant of *ORE1* and that of *ANAC046*. Recent reports have shown that *ORE1* is a miR164 target gene^22^ and its expression is enhanced by EIN3^26^. In contrast, *ANAC046* has neither miR164 target site nor EIN3 binding site, suggesting that expression of these NAC TFs controlled by different regulatory mechanism. Public database (eFP browser; http://bbc.botany.utoronto.ca/efp/cgi-bin/efpWeb.cgi) showed that these NAC TFs have different expression profiles under various senescence-promoting conditions: e.g., the expression of *ORS1* and *ANAC046* is upregulated in the dark, whereas that of *ORE1* is only slightly affected. Conversely, *ORE1* expression is upregulated by abscisic acid (ABA) treatment, whereas the expression of *ANAC046* only slightly upregulated and that of *ORS1* is unchanged. Therefore, we conclude that ANAC046, ORE1, and ORS1 share common functions but also have distinct roles and expression patterns in leaf senescence. Very recently, Sakuraba *et al*.^32^ reported that ANAC016 directly binds to the *SGR1* promoter and activates its expression during leaf senescence, whereas it does not bind to the promoters of other Chl catabolic genes, including *NYC1*, *SGR2*, and *PAO*. The result also indicates that several NAC TFs are involved in the regulation of Chl catabolic gene expression, although each has distinct regulatory roles.

As recent studies revealed, some NAC TFs form homo/hetero-dimer and influence the activity each other^33^. In addition to the direct protein-protein interaction, some NAC TFs are known to activate expression of other NAC TFs^34^,^35^. In our microarray data, some senescence-associated NAC TFs such as *ATAF1*^36^, *ANAC055*, *ANAC019*, *RD26/ANAC072*^37^, *ANAC087*^38^, and *ANAC048* were upregulated in *ANAC046* OX plants and downregulated in *ANAC046* SRDX and/or KO plants while expression of *NAP*^25^, *ORE1*^22^, *JUB1/ANAC042*^39^ and some other senescence-associated NAC TFs were not affected in these transgenic plants (Fig. S7). At least the former NAC TFs could be downstream regulators of ANAC046 or co-regulated TFs with ANAC046. On the other hand, the latter NAC TFs could be upstream of ANAC046 or independent from ANAC046. The complicated NAC-NAC regulatory circuit is unique aspect of plants and remains to be addressed in future studies.

In Arabidopsis, single mutants of *NYC1*^6^, *SGR1*^15^,^40^, *PPH*^9^, and *PaO*^10^,^11^,^40^ exhibit stay-green phenotypes with decreased levels of photosynthetic activity (termed “nonfunctional stay-green”). In contrast, KO and SRDX plants retained both Chl and photosynthetic proteins longer than the WT plants did during both age-dependent and dark-induced senescence. As judged by the CO_2_ assimilation rate, the decline in photosynthetic activity in the old leaves was much slower in KO and SRDX plants than in WT plants. We therefore consider the KO and SRDX plants to have functional stay-green phenotypes. Because a decline in photosynthetic activity is a typical symptom of leaf senescence, the results demonstrate that, besides regulating Chl degradation, ANAC046 plays important roles in other physiological events during leaf senescence, including regulation of photosynthetic activity.

Overexpression of *ANAC046* severely impaired plant growth and enhanced leaf yellowing. These phenotypes were not observed in plants overexpressing *ORE1* and *ORS1*, again showing the functional difference between ORE1 and ANAC046^20^,^23^,^24^. Furthermore, mesophyll chloroplasts of the OX plants at 40 DAS contained large starch granules at the end of the dark period, whereas the WT plants did not (Fig. 4). This suggests that the OX plants cannot degrade as much starch during the dark period as WT plants can, which may reduce carbon supply and therefore plant size becomes small. It is noteworthy that the phenotypes of loss-of-function mutants defective in starch metabolism such as *maltose excess 1* (*mex1*: maltose transporter) and *disproportionating enzyme 1* (*dpe1*: debranching enzyme)^41^,^42^ are similar to that of *ANAC046* OX plants. However, there was no significant difference in the expression levels of *MEX1* and *DPE1* between the OX and WT plants in our microarray analysis (Fig. S8). Accumulation of starch generally occurs in old leaves, although the molecular mechanism of this selectivity remains unclear^43^,^44^. It is therefore reasonable to speculate that starch accumulation is a senescence syndrome that occurs earlier in the OX plants than in the WT plants.

Plant hormones positively or negatively regulate leaf senescence by integrating developmental and environmental cues^45^. It is widely acknowledged that ABA, jasmonic acid (JA), salicylic acid (SA), and ethylene are positive regulators of leaf senescence, whereas cytokinins, gibberellins, and auxins are negative regulators. The signaling pathways of these plant hormones communicate with each other and cooperatively or antagonistically regulate senescence. To elucidate the role of each plant hormone in senescence, much effort has been made to analyze mutants defective in the signaling pathway of each hormone. Buchanan-Wollaston *et al*.^46^ have identified a set of senescence-related genes downregulated in the SA signaling–defective mutant *NahG*; many of them are upregulated during age-dependent senescence but are unaffected by dark treatment. We examined the microarray data of *ANAC046* mutants to see whether the expression of SA-dependent and developmentally regulated genes is affected by ANAC046). However, expression of these genes were only slightly affected in the OX and KO plants (Fig. S9). In contrast, nearly all senescence-related genes downregulated in both the JA signaling–defective mutant *coi1* and ethylene signaling–defective mutant *ein2*^46^ were upregulated in the OX plants and downregulated in the KO and SRDX plants (Fig. S10). Most genes dependent on the JA pathway, ethylene pathway, or both are also upregulated during dark-induced senescence^46^. These results suggest that ANAC046 plays a role in controlling developmental and dark-induced senescence downstream of JA and ethylene signaling pathways.

Recently, several TFs involved in hormonal signaling pathway have been identified as positive regulators of Chl catabolic gene expression. ABA INSENSITIVE5 (ABI5) and ENHANCED EM LEVEL (EEL) directly bind to the promoters of *SGR1*, *SGR2*, and *NYC1* in chromatin immunoprecipitation assays^47^. In Arabidopsis embryos, SGRs play a key role in degreening, which is regulated by ABA through ABI3. Direct binding of ABI3 to the promoters of *SGR1* and *SGR2* was demonstrated by gel-shift assay^48^. MYC2/3/4, a major JA signaling component, directly promote the expression of *SGR1*, *NYC1*, and *PaO*^49^. However, these TFs involved in ABA- and JA-signaling did not bind to the promoters of either *SGR1* or *NYC1* in our Y1H screening. We found that several TFs (in addition to ANAC046, ANAC087, and ANAC100) bind to the promoters of *SGR1*, *SGR2*, *NYC1*, and *PaO*, although the precise functions of these TFs remain to be elucidated. It is possible that the Chl catabolic pathway is finely tuned by multiple TFs involved in various aspects of plant growth and development.

## Conclusion

Our comprehensive Y1H screening for TFs that directly target Chl catabolic genes identified a previously uncharacterized TF, ANAC046. We showed that ANAC046 stimulates the expression not only of Chl catabolic genes but also of senescence-related genes. We also identified several TFs that directly bind the promoters of Chl catabolic genes. We assume that Chl degradation and leaf senescence are controlled by multiple TFs, which may function redundantly. Further study is needed to unravel the integration of these TFs into the molecular network that controls Chl degradation and leaf senescence.

## Methods

### Plant materials and growth conditions

*Arabidopsis thaliana* ecotype Columbia-0 was used throughout this study. The Arabidopsis transposon-tagged line SM_3_27647 from Nottingham Arabidopsis Stock Centre (NASC) was used as the *anac046* knockout mutant. Seedlings were grown on MS-agar plates in a climate chamber under fluorescent light (120 μmol m^−2^ s^−1^; 16-h light/8-h dark) at 20 °C, transferred into soil at 15 DAS, and grown in controlled-growth cabinets under the same conditions except for the temperature, which was 22/16 °C day/night). For dark treatment, at 35 DAS plants were placed in a growth cabinet at 22 °C without light.

### Plasmid construction and transformation into plants

For the Y1H experiment, the promoter region of each gene was cloned into the pDONR_P4P1R vector by Gateway BP reaction to produce entry clone (Life Technologies). Primers used for amplification of the promoter regions are listed in Table S2. The promoters were subcloned into the R4L1pDEST_HISi vectors by Gateway LR reaction (Life Technologies). Only *MES16* promoter was cloned into R4L1pDEST_HISi2 in which extra start codons between TATA-box and start codon of HIS3 are mutated. Yeast strains harboring bait constructs in their genomes were prepared from strain YM4271 by sequential insertion of promoter:*HIS3* into genomic *HIS3* loci.

To produce reporter constructs for transient effector–reporter analysis, promoter regions in the above-described entry clones were transferred into the R4L1pDEST_LUC_HSP vector^50^ by Gateway LR reaction. To produce the effector constructs, coding region of each *ANAC046* and *VAMP722* (which localizes to vacuolar membrane; negative control) was cloned in to the pDONR207 vector by Gateway BP reaction and then subcloned into the pDEST35SVP16HSPG vector, which is modified from p35SSRDXHSPG^51^, by Gateway LR reaction.

To construct *35S:ANAC046-SRDX*, the protein-coding region of *ANAC046* without the stop codon was amplified by PCR and cloned into the *Sma*I site of p35SSRDXG^52^. To construct *35S:ANAC046*, the coding region with the stop codon was amplified and cloned into the *Sma*I–*Sal*I site of p35SHSPG^51^. The transgene cassette was transferred into the T-DNA destination vector pBCKH by Gateway LR reaction. The *35S:ANAC046-SRDX* and *35S:ANAC046* constructs were transformed into Arabidopsis plants by the floral dip method^53^.

### Y1H screening

Yeast strains harboring bait constructs in their genomes were tested for the background *HIS3* gene expression by spotting liquid-grown cultures onto media with various concentrations of 3-amino-1,2,4-triazole (3-AT). After determination of the range of appropriate 3-AT concentration to suppress prey-independent growth, liquid-grown yeast strains were transformed individually with 343 mini-pools (which cover 1304 TFs) in four 96-well plates as described previously^27^. The transformed yeasts were spotted onto four sets of four square plates (three different 3-AT concentrations plus positive control) and cultured for several days. After identification of a positive mini-pool in the first screening, the positive TF in this mini-pool was identified in the second screening.

### Transient effector–reporter analysis in plants

Leaf mesophyll cells were isolated from peeled rosette leaves of 3- to 4-week-old Arabidopsis plants using the Tape Arabidopsis Sandwich method^54^. Protoplasts were prepared and transfected using the polyethylene glycol method described previously^55^. Reporter constructs containing the firefly luciferase gene under the control of the promoters of chlorophyll catabolic genes were co-transfected with the effector construct containing *ANAC046* or *VAMP722* fused with the VP16 transcriptional activation domain driven by the CaMV 35S promoter. The modified Renilla luciferase gene driven by the CaMV 35S promoter (phRLHSP) was also co-transfected as an internal control. The transfected protoplasts were incubated at 22 °C for 16–18 h in the dark. The dual-luciferase assay was carried out using the Pikka Gene Dual Assay Kit (Toyo Ink, Inc.). Reporter activity was normalized to the activity of Renilla luciferase and expressed as relative luciferase activity.

### Chlorophyll extraction and HPLC analysis

Tissues were ground into powder in liquid nitrogen and extracted with acetone. The samples (80 μl) were mixed with 20 μl of water. Pigments were analyzed by high-performance liquid chromatography (HPLC; X-LC, Jasco) using a reversed-phase column (Symmetry C8, 150 × 4.6 mm; Waters) according to Zapata *et al*.^56^.

### Western blotting

Proteins were extracted from rosette leaves according to Sakuraba *et al*.^57^. Protein extracts equivalent to 1.0 mg of leaf tissue per lane were subjected to electrophoresis on a 10% polyacrylamide gel (PAGEL; ATTO) and blotted onto a nitrocellulose membrane (GE Healthcare). Primary antibodies against PsaA, PsbA, Lhca1, Lhcb1, and RbcL were purchased from Agrisera. Anti-rabbit IgG linked to horseradish peroxidase (GE Healthcare) was used as a secondary antibody. Horseradish peroxidase activity was detected using Immobilon Western Chemiluminescent HRP Substrate (Millipore) according to the manufacturer’s protocol.

### Transmission electron microscopy (TEM)

Leaves were cut into small pieces and rapidly immersed in 0.05 M cacodylate buffer (pH 7.4) containing 2% paraformaldehyde and 2% glutaraldehyde at 4 °C overnight. After fixation, samples were rinsed 3 times with 0.05 M cacodylate buffer, followed by post-fixation with 2% osmium tetroxide in 0.05 M cacodylate buffer at 4 °C for 4 h. Samples were dehydrated in a graded ethanol series, infiltrated with propylene oxide, and finally embedded in Quetol-651 (Nisshin EM Co.). Ultrathin sections were cut with a diamond knife using an ultramicrotome (Ultracut UCT; Leica Microsystems), picked up on copper grids, and stained with uranyl acetate and lead citrate. Sections were observed under a transmission electron microscope (JEM-1200EX; JEOL Ltd) at an acceleration voltage of 80 kV. Digital images were taken with a CCD camera (Veleta; Olympus Soft Imaging Solutions GmbH).

### Ion leakage measurements

Rosette leaves were placed in a tube, immersed in 10 ml of 0.4 M mannitol solution, and shaken at room temperature for 3 h. Initial conductivity of the solution was measured using an electroconductivity meter (TOA DKK). Subsequently, the samples were boiled for 10 min, cooled down to room temperature, and measured for total conductivity. Conductivity was expressed as the percentage of the initial conductivity versus the total conductivity.

### CO_2_ assimilation rate of leaves

CO_2_ assimilation rates were determined at 20 °C using a modified portable-gas-exchange system (LI6400; Li-Cor). Whole plants were exposed to an LED light source (100 μmol photons m^−2^ s^−1^) in the chamber.

### Microarray analysis

Microarray experiments were performed using Agilent Arabidopsis (V4; 4 × 44k) microarrays according to the manufacturer’s instructions. Total RNA was extracted from leaves of WT and OX plants at 50 DAS and from leaves of WT, KO, and SRDX plants at 5 DAD using TRIzol reagent (Life Technologies) and an RNeasy Mini Kit (Qiagen). RNA integrity was evaluated using an Agilent 2100 Bioanalyzer (Agilent Technologies). Total RNA (200 ng) was used as starting material. Three biological replicates were tested by the one-color method. RNA from three independent lines of the OX and SRDX plants was used for analysis. Spot signal values were calculated by the Feature Extraction software (version 9.1; Agilent). We defined the QC value as 1 when a spot passed the “FeatNonUnifOL” filter and as 2 when the spot further passed the “FeatPopnOL” filter. We defined the detection value as 1 when a spot passed the “IsPosAndSignif” filter and as 2 when the spot further passed the “IsWellAboveBG” filter. All signal values were divided by the median value among spots with QC = 2 followed by quantile normalization^58^ using all previously obtained microarray data in our group to make each signal distribution the same. Spot-to-gene conversion was accomplished based on a table provided by The Arabidopsis Information Resource (ftp://ftp.Arabidopsis.org/home/tair/Microarrays/Agilent/agilent_array_elements-2010-12-20.txt).

The upregulated genes in OX, KO, and SRDX plants were defined as top 200 upregulated genes with the false discovery rate <0.15; the latter was calculated using the QVALUE software with default settings^59^.

### Quantitative real-time PCR analysis

The transcript levels of *ANAC046* was analyzed by quantitative real-time PCR (RT-qPCR) with the SYBR Premix Ex Taq II polymerase (TaKaRa) and Thermal Cycler Dice Real Time System TP800 (TaKaRa), according to the manufacturers’ instructions. The expression level of *actin* was used to normalize the transcript levels of each sample. Primers used for RT-qPCR analysis were shown in Table S4.

## Additional Information

**Accession codes:** Sequence data from this article can be found in The Arabidopsis Information Resource under the following accession numbers: *CLH1* (AT1G19670), *CLH2* (AT5G43860), *CYP89A9* (AT3G03470), *HCAR* (AT1G04620), *NOL* (AT5G04900), *NYC1* (AT4G13250), *PAO* (AT3G44880), *PPH* (AT5G13800), *RCCR* (AT4G37000), *SGR1/NYE1* (AT4G22920), *SGR2* (AT4G11910), *ANAC046* (AT3G04060), *ANAC087* (AT5G18270), *ORE1* (AT5G39610), *ORS1* (AT3G29035), *ANAC079* (AT5G07680), and *ANAC100* (AT5G61430). Microarray data were deposited in the National Center for Biotechnology Information Gene Expression Omnibus (http://www.ncbi.nlm.nih.gov/geo/) under accession number GSE71767.

**How to cite this article**: Oda-Yamamizo, C. *et al*. The NAC transcription factor ANAC046 is a positive regulator of chlorophyll degradation and senescence in Arabidopsis leaves. *Sci. Rep*. **6**, 23609; doi: 10.1038/srep23609 (2016).

## Supplementary Material

Supplementary Information

## Figures and Tables

**Figure 1:**
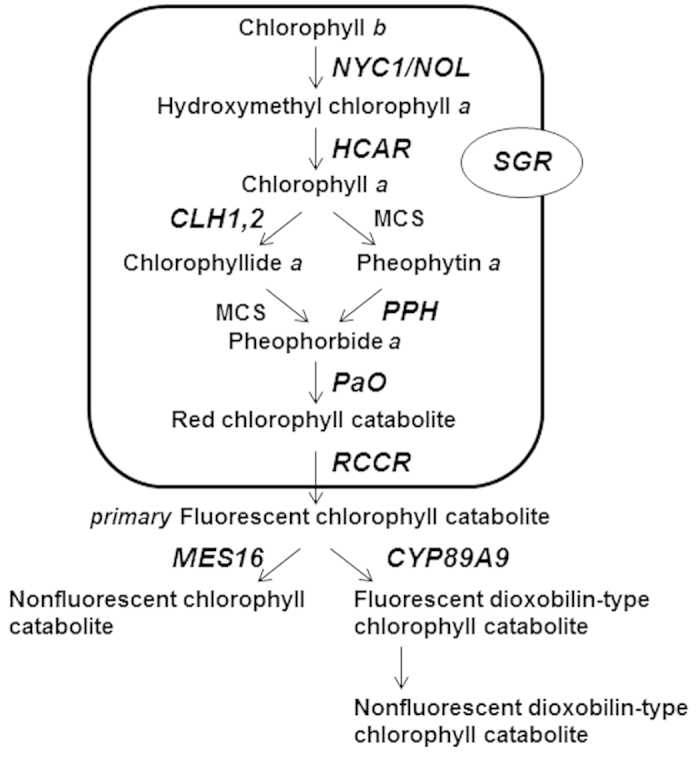
Schematic representation of the chlorophyll catabolic pathway in Arabidopsis. Genes (italicized) and encoded enzymes/proteins are as follows: *NYC1/NOL*, chlorophyll *b* reductase; *HCAR*, hydroxymethyl chlorophyll *a* reductase; *PPH*, pheophytinase; *PaO*, pheophorbide *a* oxygenase; *RCCR*, red chlorophyll catabolite reductase; *MES16*, methylesterase 16; *CYP89A9*, cytochrome P450 enzyme; *SGR*, STAY-GREEN. MCS, metal-chelating substance.
