# Corrigendum: The NAC transcription factor ANAC046 is a positive regulator of chlorophyll degradation and senescence in Arabidopsis leaves

**DOI:** 10.1038/srep35125

**Published:** 2016-10-19

**Authors:** Chihiro Oda-Yamamizo, Nobutaka Mitsuda, Shingo Sakamoto, Daisuke Ogawa, Masaru Ohme-Takagi, Akemi Ohmiya

Scientific Reports 6: Article number: 2360910.1038/srep23609; published online: 03 29 2016; updated: 10 19 2016

The original Supplementary Information published with this Article contained an error in the order of the Figures. Figures S9 and S10 were published as Figures S10 and S9 respectively. This error has now been corrected in the Supplementary Information that now accompanies the Article.

