# Supplementary material for: The NAC transcription factor ANAC046 is a positive regulator of chlorophyll degradation and senescence in Arabidopsis leaves
Source: Sci Rep. 2016 Mar 29;6:23609. doi: 10.1038/srep23609 (PMC4810360; doi:10.1038/srep23609)
Supplement: Supplementary Information [file srep23609-s1.pdf]

The NAC transcription factor ANAC046 is a positive regulator of chlorophyll degradation and senescence in Arabidopsis leaves

Chihiro Oda-Yamamizo, Nobutaka Mitsuda, Shingo Sakamoto, Daisuke Ogawa, Masaru Ohme-Takagi and Akemi Ohmiya

Supplementary Figure S1

Locus	Alias	TF classification	CYP89 A9	HCAR	RCCR	MES16	PPH	NOL	NYC1	PaO	SGR1	SGR2
AT3G12680	HUA1	C3HZnF	0	0	0	0	0	0	0	0	1	0
AT3G02830	PNT1, ZFN1	C3HZnF	0	0	0	0	0	0	0	0	1	0
AT3G49610		B3	0	0	0	0	0	0	0	0	2	0
AT1G32640	JIN1, JAI1, ZBF1,	bHLH	0	0	0	0	0	0	0	2	0	0
AT2G21230	AtbZIP30	bZIP	0	0	0	0	0	0	0	2	0	1
AT4G38900	AtbZIP29	bZIP	0	0	0	0	0	0	0	3	0	1
AT1G75390	ATBZIP44	bZIP	0	0	0	0	0	1	0	0	0	0
AT1G32150	AtbZIP68	bZIP	0	0	0	0	0	3	2	0	0	0
AT2G46270	GBF3	bZIP	0	0	0	0	0	3	3	0	0	0
AT4G36730	AtGBF1, GBF1	bZIP	0	0	0	0	0	3	3	0	0	0
AT1G12260	ANAC007, VND4,	NAC	0	0	0	0	0	0	1	0	0	0
AT1G32510	ANAC011	NAC	0	0	0	0	0	0	1	0	0	0
AT5G46590	ANAC096	NAC	0	0	0	0	0	0	1	0	0	0
AT1G34180	ANAC016	NAC	0	0	0	0	0	0	2	0	0	0
AT3G10500	NTL4, ANAC053,	NAC	0	0	0	0	0	0	3	0	0	0
AT3G03200	ANAC045	NAC	0	0	0	0	0	0	2	0	0	1
AT3G17730	ANAC057	NAC	0	0	0	0	0	0	3	0	0	1
AT4G10350	BRN2, NST4,	NAC	0	0	0	0	0	0	3	0	0	1
AT1G76420	NAC368, CUC3,	NAC	0	0	0	0	0	0	0	0	1	1
AT3G15170	ATNAC1, CUC1,	NAC	0	0	0	0	0	0	0	0	1	1
AT1G56010	NAC1, ANAC021,	NAC	0	0	0	0	0	0	0	0	3	1
AT1G33280	BRN1, ANAC015,	NAC	0	0	0	0	0	0	1	0	0	3
AT2G18060	ANAC037, VND1	NAC	0	0	0	0	0	0	0	0	0	1
AT1G79580	URP7, ANAC033,	NAC	0	0	0	0	0	0	0	0	0	1
AT1G52880	ATNAM, NAM,	NAC	0	0	0	0	0	0	0	0	0	1
AT1G52890	ANAC019	NAC	0	0	0	0	0	0	0	0	0	1
AT1G61110	ANAC025	NAC	0	0	0	0	0	0	0	0	0	2
AT5G49420	ATAF1, ANAC002	NAC	0	0	0	0	0	0	0	0	0	3
AT2G24430	ANAC039,	NAC	0	0	0	0	0	0	0	0	0	3
AT1G65910	ANAC028	NAC	0	0	0	0	0	0	2	0	0	3
AT1G71930	VND7, ANAC030	NAC	0	0	0	0	0	0	1	0	0	1
AT1G54330	ANAC020	NAC	0	0	0	0	0	0	3	0	0	3
AT5G53950	ATCUC2, CUC2,	NAC	0	0	0	0	0	0	1	0	0	1
AT3G18400	ANAC058	NAC	0	0	0	0	0	0	0	2	0	1
AT4G28530	ANAC074	NAC	0	0	0	0	0	0	2	2	0	3
AT3G15500	ATNAC3,	NAC	0	0	0	0	0	0	0	2	0	3
AT5G07680	ANAC079,	NAC	0	0	0	0	0	0	0	3	1	3
AT5G39610	ORE1, AtNAC2,	NAC	0	0	0	0	0	0	1	3	1	3
AT3G04060	ANAC046	NAC	0	0	0	0	0	0	3	3	3	3
AT5G18270	ANAC087	NAC	0	0	0	0	0	0	3	3	3	3
AT5G61430	ANAC100,	NAC	0	0	0	0	0	0	3	3	3	3
AT1G76500	SOB3, AHL29	AT-hook	3	2	0	0	0	0	0	0	0	0
AT1G20900	ORE7, AHL27,	AT-hook	3	3	0	0	0	0	0	0	0	0
AT3G55560	AHL15, AGF2	AT-hook	3	3	0	0	0	0	0	0	0	0
AT3G04570	AHL19	AT-hook	0	3	0	0	0	0	0	0	0	0
AT1G14490	AHL28	AT-hook	0	3	0	0	0	0	0	0	0	0
AT2G42940	TEK, AHL16	AT-hook	0	1	0	0	0	0	0	0	0	0
AT4G35390	AHL25, AGF1	AT-hook	0	2	0	0	0	0	0	0	0	0
AT4G22810	AHL24	AT-hook	0	2	0	0	0	0	0	0	0	0
AT4G12050	AHL26	AT-hook	0	2	0	0	0	0	0	0	0	0
AT5G49700	AHL17	AT-hook	1	3	0	0	0	0	0	0	0	0
AT5G21120	EIL2	EIL	0	0	0	3	0	0	0	0	0	0
AT5G39750	AGL81, EMB3008	MADS	0	0	1	2	0	0	0	0	0	0

Supplementary Figure S1. Transcription factors (TFs) that bind to the promoter regions of Chl catabolic genes as detected by Y1H screening. The numbers in the table indicate the signal strength (0=weak to 3=strong) of each clone judged by visual inspection.

Supplementary Figure S2

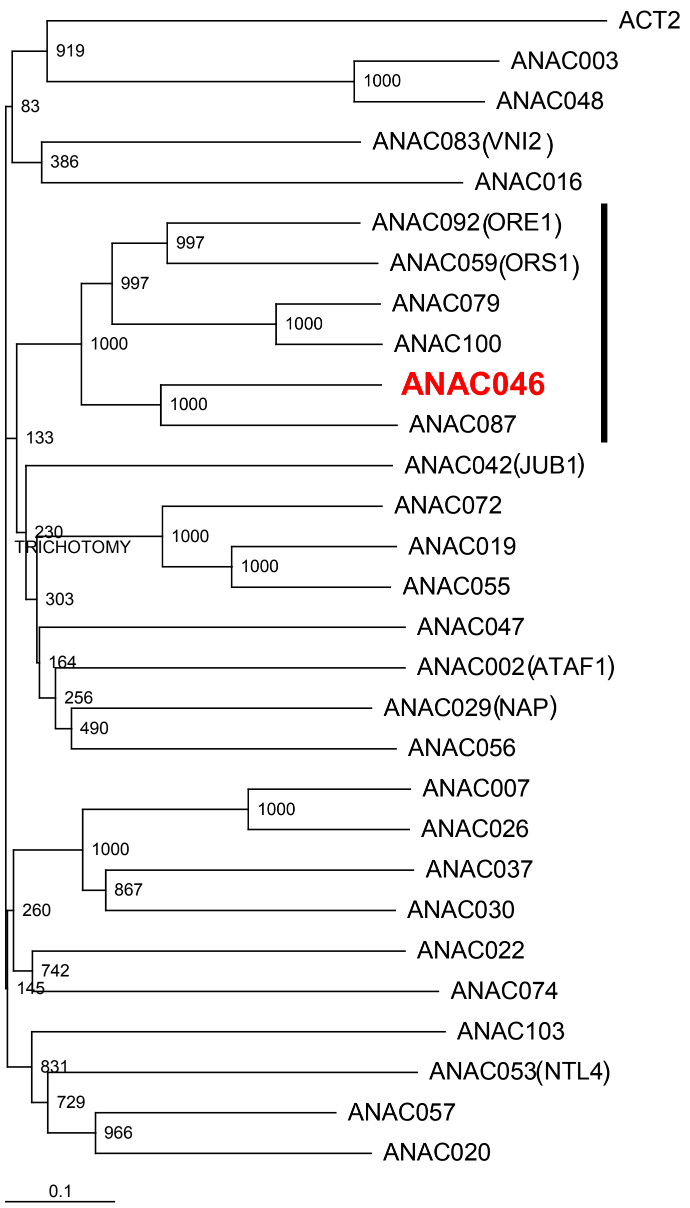

Supplementary Figure S2. Phylogenic tree based on the entire amino acid sequences of senescence-inducible NAC transcription factors in Arabidopsis. Phylogenetic relationship was deduced by using the neighbor-joining method. The frequency with which a given branch appeared in 1000 bootstrap replications is indicated at the nodes. Vertical bar indicates the NAM subgroup. Scale bar indicates 0.1 amino acid substitutions per site.

Supplementary Figure S3

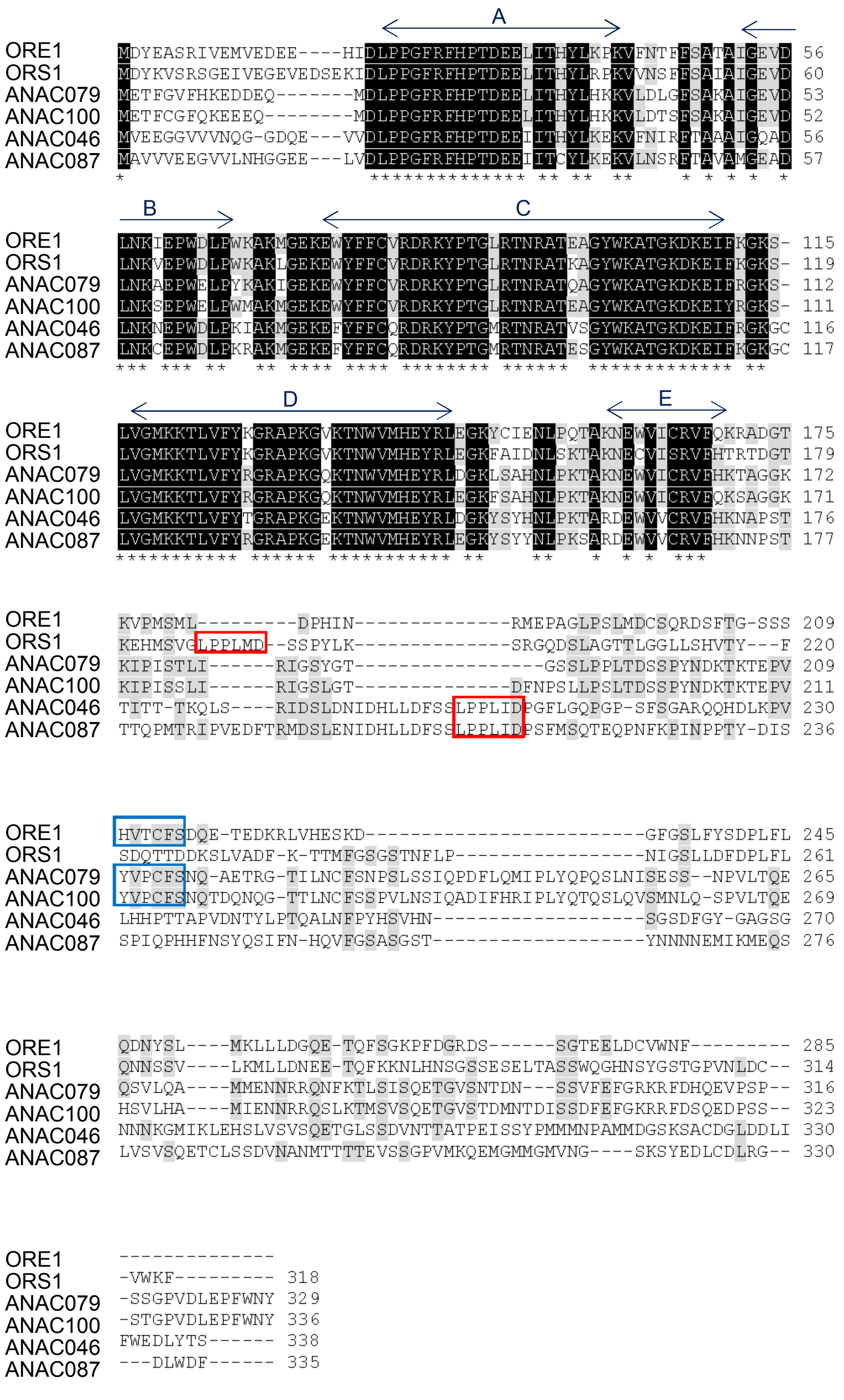

Supplementary Figure S3. Alignment of deduced amino acid sequences of transcription factors of the NAM subgroup. Multiple sequence alignment was performed with ClustalW. Amino acids completely conserved within the NAM subgroup are shaded in black and those conserved in more than three sequences are shaded in gray. Red squares indicate motif conserved within the NAM subgroup and blue squares indicate sequences corresponding to the predicted miR164 target sites. A–E, highly conserved subdomains within the NAC domain.

Supplementary Figure S4

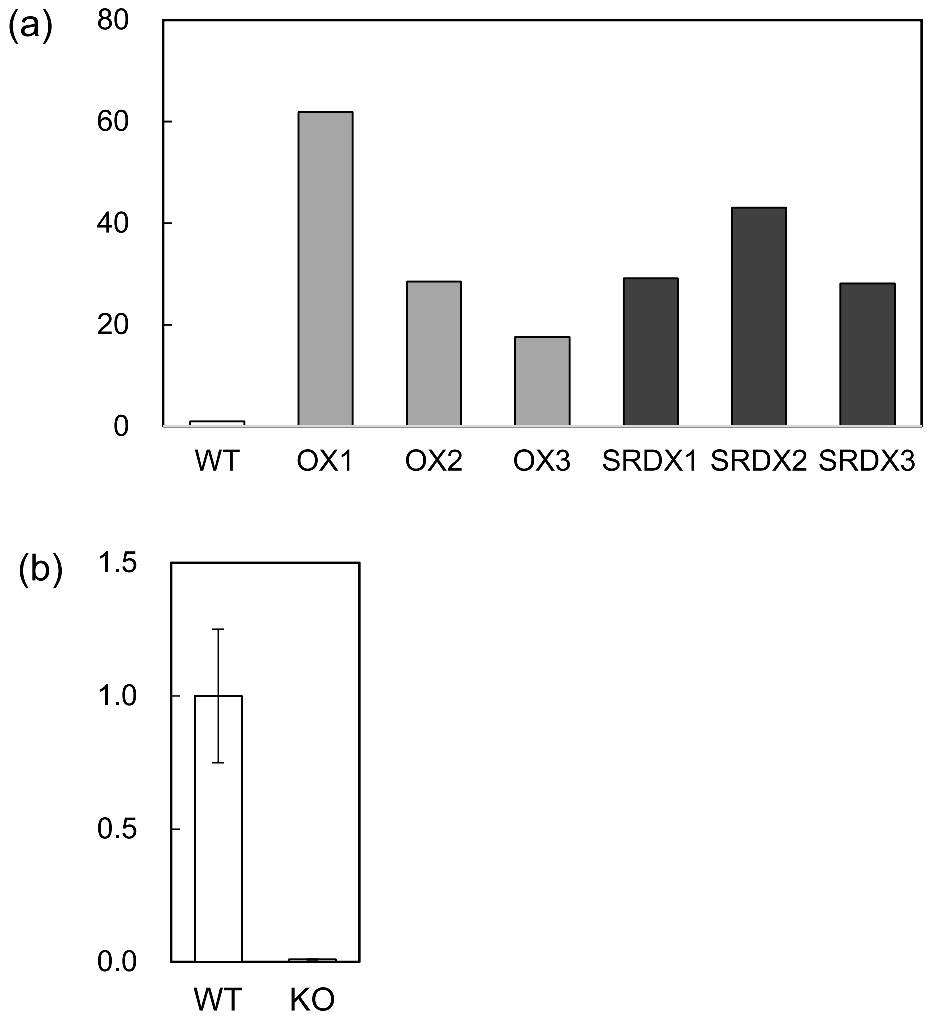

Supplementary Figure S4. Expression of ANAC046 in OX, SRDX, and KO plants. (a) Expression of *ANAC046* in OX and SRDX plants. Three indipendent transgenic lines were analyzed. The data represent values of single replicate. (b) Expression of *ANAC046* in KO plants. Data are means ±SD of three technical replicates. Relative expression was analyzed by quantitative real-time PCR using *ANAC046* specific primers (Table S4).

Supplementary Figure S5

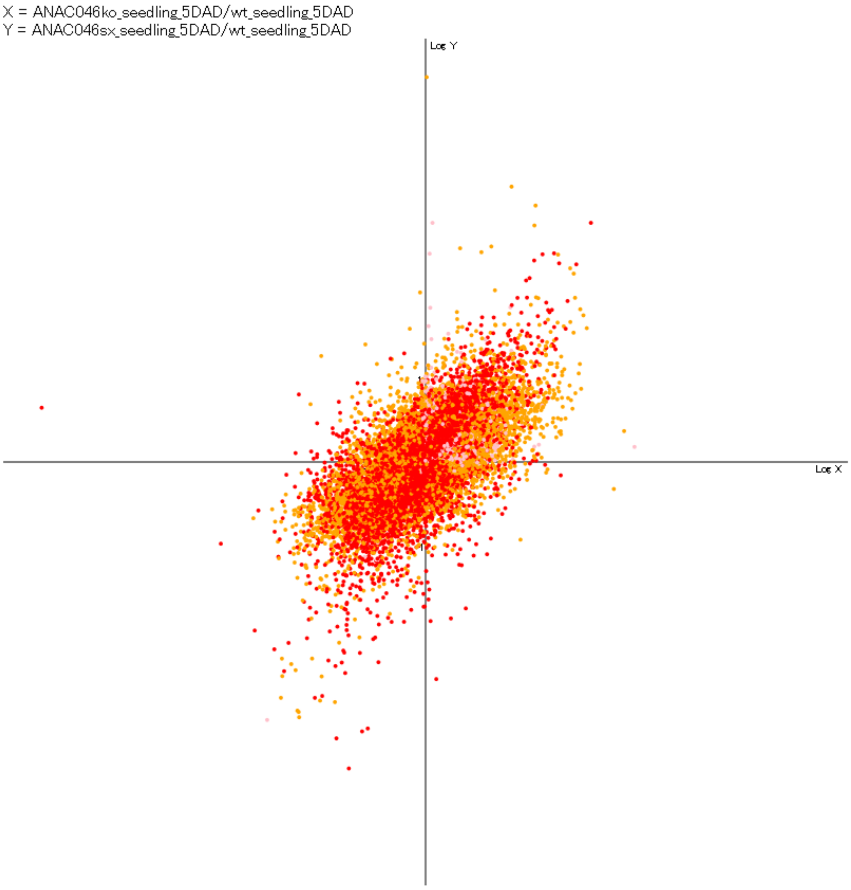

Supplementary Figure S5. Scatter plot of log values of fold changes in the expression of all genes in a KO plant (*X* axis) and SRDX plant (*Y* axis). Fold changes were well correlated between the two plants. Red, orange, and pink dots represent genes expressed at a high level (average detection value in KO and SRDX plants ≥ 1.5), marginal level (average detection value ≥ 0.5), and low level (average detection value < 0.5), respectively.

Supplementary Figure S6

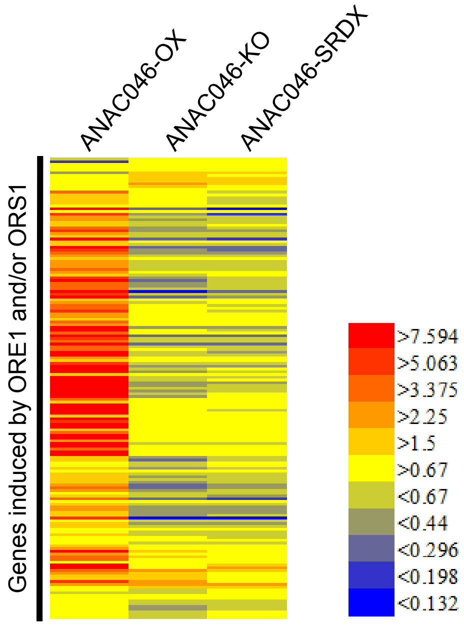

Supplementary Figure S6. Expression of ORE1- and/or ORS1-inducible genes in the *ANAC046* OX, KO, and SRDX plants. Color indicates level of fold change in each transgenic/mutant plant against wild-type plant.. The list of ORE1- and ORS1-inducible genes was obtained from Balazadeh et al. (2010) [20], Balazadeh et al. (2011)[24], and Matallana-Ramirez et al. (2013).

Supplementary Figure S7

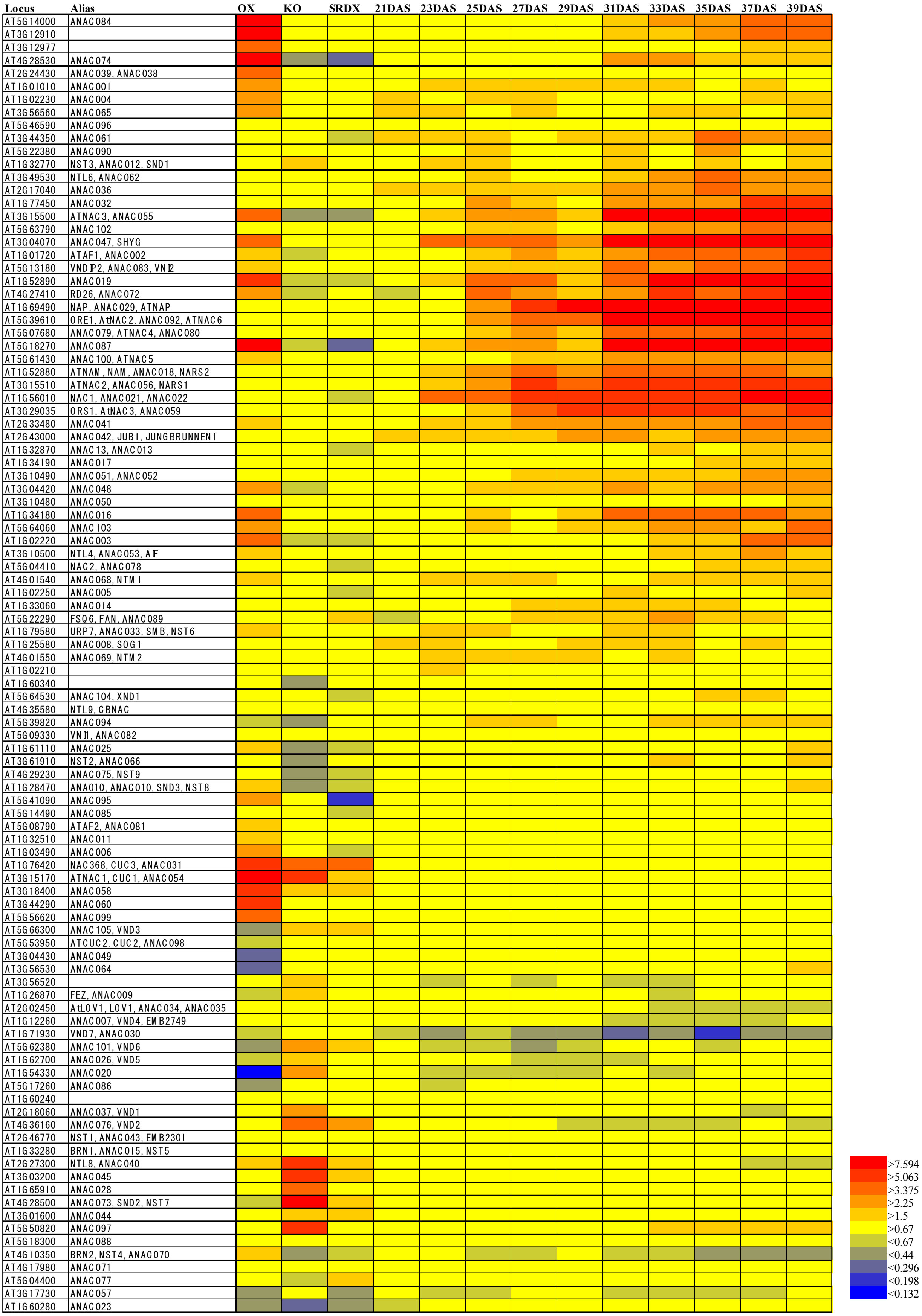

Supplementary Figure S7. Fold change of NAC transcription factor genes in the ANAC046OX, KO, and SRDX plants in addition to fold change in afternoon of 21, 23, 25, 27, 29, 31, 33, 35, 37, and 39 days after sowing (DAS) over one in afternoon of 19DAS.

Supplementary Figure S8

Locus	Alias	OX	KO	SRDX
AT5G64860	DPE1			
AT5G17520	MEX1			

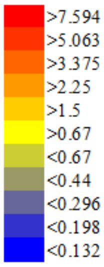

Supplementary Figure S8. Expression of *DPE1* and *MEX1* in the *ANAC046* OX, KO, and SRDX plants relative to WT plants.

Supplementary Figure S9

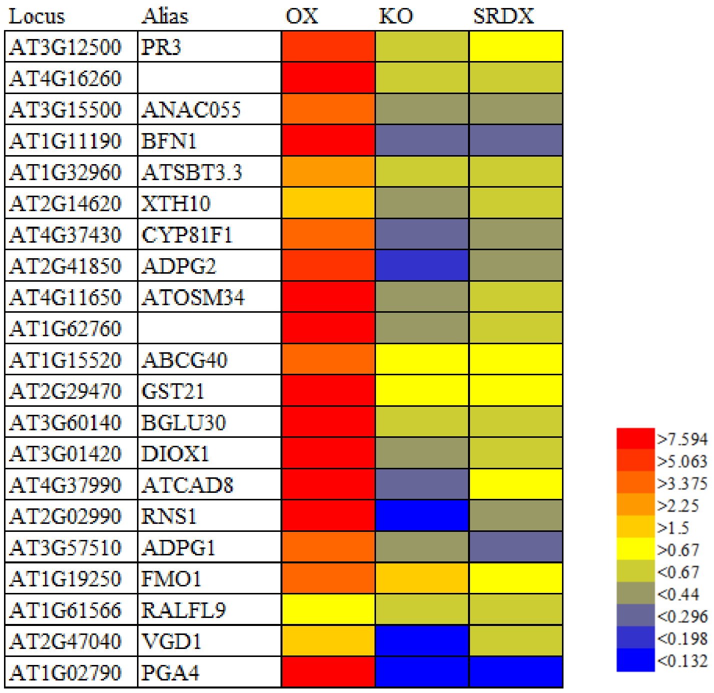

Supplementary Figure S9. Expression of senescence-inducible genes dependent on salicyclic acid signaling in the *ANAC046* OX, KO, and SRDX plants. The list of senescence-inducible genes downregulated in the *NahG* plants (deficient in salicyclic acid signaling) was obtained from Buchanan-Wollaston et al. (2005). The expression of these genes was upregulated during age-dependent senescence but not dark-induced senescence (Buchanan-Wollaston et al., 2005).

Supplementary Figure S10

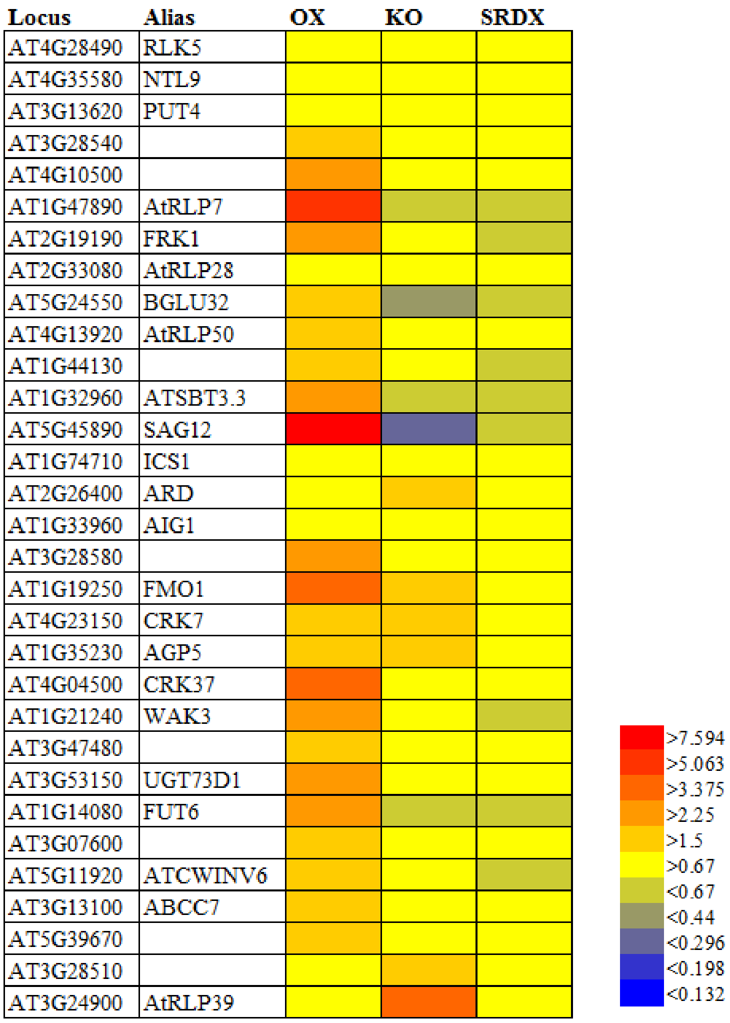

Supplementary Figure S10. Expression of senescence-inducible genes dependent on jasmonic acid signaling, ethylene signaling, or both in the *ANAC046* OX, KO, and SRDX plants. The list of senescence-inducible genes downregulated in the *coi1* mutant (deficient in jasmonic acid signaling) and *ein2* mutant (deficient in ethylene signaling) was obtained from Buchanan-Wollaston et al. (2005). Color indicates level of fold change in each transgenic/mutant plant against wild-type plant.

Supplementary Table S1

Detailed fold change of ORE1- and/or ORS1-inducible genes in the ANAC046OX, KO, and SRDX plants shown in Supplementary Figure 5.

Locus	Alias	OX	KO	SRDX
AT3G59900	ARGOS	0.43	0.81	1.31
AT1G49500		0.63	0.72	1.29
AT1G13080	CYP71B2	0.83	1.01	1.03
AT2G21220	SAUR12	0.18	1.21	1.05
AT4G35770	ATSEN1, DIN1, SEN1	0.91	1.02	0.99
AT1G63260	TET10	0.93	1.19	0.96
AT2G41730		0.79	1.19	0.95
AT3G48360	ATBT2, BT2	0.48	1.47	1.44
AT1G49230	AtATL78	0.42	1.85	1.91
AT2G28200		0.67	1.58	1.4
AT1G58170		0.95	1.5	1.83
AT3G10525	LGO, SMR1	0.84	1.77	1.71
AT5G62960		0.76	4.68	3.78
AT5G23750		0.73	2.29	1.52
AT5G64620	ATC/VIF2, C/VIF2	0.83	1.55	1.3
AT1G08810	ATMYB60	0.96	1.44	1.16
AT4G04490	CRK36	4.5	0.95	0.5
AT1G05450		5.12	0.79	0.31
AT4G34630		1.71	0.98	0.7
AT1G72790		1.62	0.91	0.53
AT1G55760		1.78	0.92	0.58
AT4G26470		1.63	0.89	0.64
AT5G50260	CEP1	129.79	0.23	0.01
AT5G18640		1.39	0.87	0.78
AT4G18425		18.16	0.21	0.08
AT2G32150		1.49	0.8	0.69
AT1G26820	RNS3	5.42	0.35	0.14
AT1G79520		2.75	0.52	0.6
AT4G13250	NYC1, NYC	3.12	0.46	0.56
AT5G40690		3.22	0.43	0.53
AT3G49210		2.16	0.57	0.65
AT2G25625		3.23	0.39	0.5
AT3G49620	DIN11	1.78	0.66	0.69
AT1G73750		3.42	0.42	0.48
AT5G54570	BGLU41	5.95	0.35	0.38
AT1G07610	MT1C	2.77	0.54	0.55
AT3G62590		4.3	0.44	0.44
AT2G26670	GUN2, ATHO1, TED4, HY6, HY1	2.2	0.65	0.63
AT1G73000	RCAR13, PYL3	20.81	0.2	0.18
AT5G51260		1.65	0.76	0.73
AT5G43060	RD21B	2.09	0.6	0.55
AT2G14095		11.18	0.22	0.15
AT1G11190	ENDO1, BFN1	9.63	0.25	0.2
AT5G04200	AtMCP2f, ATMC9	7.44	0.3	0.25
AT2G25940	ALPHAVPE, ALPHA-VPE	4.57	0.37	0.35
AT1G02660		3.16	0.55	0.45
AT5G25820		4.02	0.53	0.49
AT3G61960		1.74	0.79	0.73
AT2G34930		2.95	0.66	0.57
AT1G19200		3.02	0.56	0.59
AT3G25290		3.06	0.52	0.61
AT1G53100		1.79	0.77	0.8
AT4G22920	ATNYE1, SGR1, STAY-GREEN1	4.3	0.53	0.55
AT1G09240	ATNAS3	2.46	0.67	0.67
AT3G01830		2.08	0.61	0.73
AT2G31945		5.51	0.32	0.49
AT2G02990	RNS1, ATRNS1	19.56	0.13	0.35
AT4G29570		10.66	0.23	0.45
AT1G02470		4.11	0.41	0.64
AT3G21520	AtDMP1	4.79	0.4	0.63
AT1G74010		4.39	0.45	0.65
AT1G56060		2.1	0.65	0.78
AT5G13170	SAG29, SWEET15, AtSWEET15	47.51	0.12	0.27
AT3G51090		6.29	0.47	0.57
AT1G09380	UMAMIT25	23.72	0.22	0.37
AT5G13180	VNDIP2, ANAC083, VNI2	1.56	0.82	0.87
AT4G18980	AtS40-3	8.57	0.38	0.59
AT1G45145	TRX-h5, LIV1, ATTRX5, ATH5	2.73	0.47	0.88
AT1G55740	RS1, ATSIP1	3.98	0.93	0.65
AT2G17710		3.55	0.88	0.68
AT5G16570	GLN1;4	5.48	0.84	0.59
AT3G14360		3.55	0.82	0.65
AT5G64120	AtPRX71	2.34	0.91	0.73
AT1G23330		3.24	0.81	0.74
AT2G32680	AtRLP23	2.01	0.89	0.82
AT1G15520	ATABCG40, ABCG40, PDR12, ATPDR12	3.66	0.79	0.7
AT5G63970	RGLG3	6.41	0.72	0.71
AT3G56170	CAN1, CAN	2.43	0.85	0.86
AT1G20180		149.28	0.38	0.33
AT1G15380	GLYI4	11.46	0.72	0.61
AT1G32350	AOX1D	4.74	0.79	0.71
AT2G29460	GST22, ATGSTU4	2.92	0.89	0.78
AT4G18550	AtDSEL	37.09	0.26	0.27
AT2G44010		6.79	0.51	0.49
AT5G13820	HPPBF-1, ATTBP1, ATBP-1, ATBP1, TBP1	4.25	0.66	0.52
AT3G52780	ATPAP20, PAP20	41.46	0.29	0.23
AT2G30770	CYP71A13	5.25	0.6	0.5
AT1G08920	ESL1	4.48	0.63	0.55
AT5G38710		4.79	0.68	0.6
AT2G46950	CYP709B2	20.52	0.45	0.33
AT4G16260		11.27	0.48	0.6
AT3G01420	PADOX-1, ALPHA-DOX1, DIOX1	22.85	0.36	0.46
AT4G01870		3.04	0.72	0.72
AT3G05180		1.98	0.8	0.82
AT3G45010	SCPL48	6.24	0.55	0.55
AT4G04460		41.86	0.39	0.35
AT2G05910		9.7	0.55	0.55
AT4G25000	AMY1, AMY3, ATAMY1	8.59	0.49	0.89
AT5G45630		27.38	0.34	0.6
AT2G33480	ANAC041	2.03	0.78	0.91
AT1G17020	ATSRG1, SRG1	17.62	0.48	0.66
AT4G11650	ATOSM34	52.9	0.34	0.55
AT4G19810	ChiC	8.21	0.57	0.71
AT1G68450	PDE337	129.66	0.3	0.43
AT3G61930		27.97	0.44	0.56
AT5G39520		13.2	0.56	0.64
AT2G44380		2.31	0.82	0.88

Locus	Alias	OX	KO	SRDX
AT4G19810	ChiC	8.21	0.57	0.71
AT1G68450	PDE337	129.66	0.3	0.43
AT3G61930		27.97	0.44	0.56
AT5G39520		13.2	0.56	0.64
AT2G44380		2.31	0.82	0.88
AT1G62760		169.14	0.38	0.57
AT1G74000	SS3	15.14	0.56	0.68
AT2G38240		27.16	0.42	0.74
AT1G80840	ATWRKY40, WRKY40	3.44	1.11	0.75
AT5G61600	ERF104	3.59	1.03	0.71
AT1G09560	PDGLP1, GLP5	2.03	1.06	0.79
AT4G25810	XTH23, XTR6	14.07	1.34	0.72
AT2G35980	NHL10, YLS9, ATNHL10	8.29	1.24	0.88
AT2G45220	PME17	37.41	0.9	0.64
AT4G39670		9.85	0.95	0.71
AT3G61190	BAP1	12.02	1	0.74
AT5G01520	AtAIRP2, AIRP2	2.06	1.01	0.9
AT5G14000	ANAC084	16.18	1.05	0.81
AT1G74590	ATGSTU10	7.43	1.05	0.83
AT3G21840	ASK7	19.22	1.02	0.87
AT1G79320	AtMCP2c, ATMC6	7.97	0.8	0.82
AT2G29470	GST21, ATGSTU3	19.25	0.7	0.8
AT3G06210		2.15	0.89	0.95
AT1G80450		3.85	0.89	0.84
AT5G13080	ATWRKY75, WRKY75	17.61	0.78	0.73
AT3G13950		12.85	0.86	0.77
AT3G48580	XTH11	16.09	0.86	0.69
AT3G13672	SINA2	3.37	0.87	0.83
AT2G32660	AtRLP22	38.7	0.6	0.58
AT2G23110		28.11	0.93	1.04
AT3G22600	LTPG5	23.53	0.88	0.99
AT3G11340	UGT76B1	3.52	1.04	1.05
AT1G69930	ATGSTU11	27.31	1.18	1.1
AT2G47950		15.12	0.74	1.1
AT2G01890	PAP8, ATPAP8	1.73	0.37	0.75
AT1G66400	CML23	1.72	0.3	0.55
AT5G02420		2.1	0.26	0.54
AT4G21980	ATG8A, APG8A	1.29	0.47	0.68
AT5G59845		1.23	0.47	0.72
AT1G12640	LPCAT1, AtLPLAT1	1.56	0.58	0.62
AT3G06420	ATG8H	2.11	0.4	0.45
AT5G59840		1.31	0.72	0.78
AT4G25700	BCH1, B1, CHY1, BETA-OHASE1 ,BETA-OHASE 1	1.66	0.52	0.6
AT5G54870		2.24	0.37	0.45
AT3G16440	ATMLP-300B, MEE36	6.53	0.1	0.16
AT5G38610		1.83	0.52	0.59
AT3G23920	AtBAM1, BAM1, BMY7, TR-BAMY	2.19	0.41	0.52
AT3G21550	AtDMP2	3.32	0.25	0.35
AT3G05400		3.64	0.23	0.4
AT1G55265		1.39	0.7	0.8
AT1G74020	SS2	2.33	0.42	0.6
AT1G29160	AtDof1. 5	1.46	0.66	0.62
AT3G29250	AtSDR4	2.15	0.45	0.41
AT1G55240		4.21	0.26	0.17
AT5G08480		5.39	0.21	0.15
AT5G67370	CGLD27	1.41	0.75	0.77
AT3G19900		1.94	0.53	0.56
AT1G19850	IAA24, ARF5, MP	2.95	0.34	0.38
AT1G48260	CIPK17, SnRK3.21	2.9	0.39	0.39
AT4G34320		10.17	0.16	0.11
AT5G01820	PKS24, CIPK14, ATSR1, SnRK3.15, ATCIPK14	1.79	0.33	0.43
AT1G47128	RD21A, RD21	1.61	0.42	0.55
AT5G56540	ATAGP14, AGP14	5.68	0.04	0.11
AT1G23040		1.43	0.55	0.54
AT1G28470	ANA010, ANAC010, SND3, NST8	1.64	0.42	0.48
AT3G26100		1.92	0.33	0.37
AT5G26940	DPD1	1.55	0.45	0.49
AT4G18910	ATNLM2, NIP1;2, NLM2	1.11	0.76	0.78
AT5G56980		1.31	0.53	0.55
AT5G14420	RGLG2	1.52	0.58	0.51
AT1G02640	ATBXL2, BXL2	1.5	0.6	0.49
AT4G12580		1.38	0.67	0.53
AT5G49710		1.1	0.82	0.74
AT5G07560	ATGRP20, GRP20	1.11	0.66	0.63
AT1G73330	ATDR4	1.57	0.41	1.14
AT4G15700		1.93	0.72	1.48
AT4G21850	ATMSRB9	2.46	1.31	1.03
AT4G16620	UMAMIT8	13.33	1.57	1.18
AT3G15810		1.57	1.08	1.06
AT3G25870		2.9	1.22	0.99
AT1G19250	FMO1	4.62	1.74	0.82
AT4G27280		3.69	1.45	0.79
AT5G17450	HIPP21	1.44	1.14	0.92
AT4G15680		17.78	1.11	1.81
AT1G19610	LCR78, PDF1.4	7.83	0.85	2.02
AT3G21830	ASK8	15.32	1.26	3.2
AT5G23530	ATCXE18	3.51	2.81	1.52
AT3G21850	ASK9	2.47	2.08	1.38
AT5G57560	XTH22, TCH4	1.91	1.72	1.2
AT2G16890		1.84	1.67	0.88
AT2G13810	AtALD1, EDTS5, ALD1	1.86	1.53	1
AT3G21860	ASK10	7.03	2.77	1.14
AT2G40230		2.74	2.6	2.81
AT4G30670		2.16	2.24	1.73
AT4G10850	SWEET7, AtSWEET7	1.4	1.72	1.5
AT3G19030		0.75	0.45	1.19
AT2G40670	ARR16	0.5	0.45	0.75
AT1G26920		0.76	0.8	0.84
AT5G39610	ORE1, AtNAC2, ANAC092, ATNAC6	0.67	0.71	0.67
AT2G42890	AML2, MEI2	0.94	0.69	0.68
AT1G10060	BCAT1, ATBCAT-1	0.88	0.54	0.56
AT3G14770	SWEET2, AtSWEET2	0.92	0.57	0.64
AT2G38480		0.75	0.41	0.49
AT5G04390		0.95	0.87	0.85
AT2G39890	ATPROT1, ProT1	1.07	0.39	0.52
AT2G25735		0.97	0.48	0.71
AT2G15830		0.96	0.46	0.76

Supplementary Table S2

Primers used for vector construction for Y1H screening and transient effector–reporter analysis

Name	Sequence (5′→3′)
CLH1-pDONR-F	GGGGACAACTTTGTATAGAAAAGTTGTTCTCTGTTTTTCT
CLH1-pDONR-R	GGGGACTGCTTTTTTGTACAAACTTGGTTGTATTAAACTA
CLH2-pDONR-F	GGGGACAACTTTGTATAGAAAAGTTGGACTCATCAGAGTG
CLH2-pDONR-R	GGGGACTGCTTTTTTGTACAAACTTGGTTTTTTTGTTCTT
CYP89A9-pDONR-F	GGGGACAACTTTGTATAGAAAAGTTGCATTTGGGAAATTT
CYP89A9-pDONR-R	GGGGACTGCTTTTTTGTACAAACTTGGTATCTTTTTTTAG
HCAR-pDONR-F	GGGGACAACTTTGTATAGAAAAGTTGCGGTGGTGAAAATG
HCAR-pDONR-R	GGGGACTGCTTTTTTGTACAAACTTGGATCTTTTAGGAAC
MES16-pDONR-F	GGGGACAACTTTGTATAGAAAAGTTGTCCGGTGAGATTCG
MES16-pDONR-R	GGGGACTGCTTTTTTGTACAAACTTGGTGCATGAATCTTG
NOL-pDONR-F	GGGGACAACTTTGTATAGAAAAGTTGTGAATCTCTGTAGG
NOL-pDONR-R	GGGGACTGCTTTTTTGTACAAACTTGGTACAGAAGCTCAA
NYC1-pDONR-F	GGGGACAACTTTGTATAGAAAAGTTGTTATTGTGTCACAG
NYC1-pDONR-R	GGGGACTGCTTTTTTGTACAAACTTGGAGCTATGGAAGAA
PAO-pDONR-F	GGGGACAACTTTGTATAGAAAAGTTGTCTCTTTCGTTTAT
PAO-pDONR-R	GGGGACTGCTTTTTTGTACAAACTTGGTTTTCTAGTTTGG
PPH-pDONR-F	GGGGACAACTTTGTATAGAAAAGTTGTGTCTAAGTTATTA
PPH-pDONR-R	GGGGACTGCTTTTTTGTACAAACTTGGAAGCGAGGAAAAA
RCCR-pDONR-F	GGGGACAACTTTGTATAGAAAAGTTGAGAACTCTTTATCA
RCCR-pDONR-R	GGGGACTGCTTTTTTGTACAAACTTGGTTTTTATGTGGCT
SGR1-pDONR-F	GGGGACAACTTTGTATAGAAAAGTTGGAAAGAAGAAGAAA
SGR1-pDONR-R	GGGGACTGCTTTTTTGTACAAACTTGGCTCTGCTCTCTTG
SGR2-pDONR-F	GGGGACAACTTTGTATAGAAAAGTTGGTTAATCGAGTTGG
SGR2-pDONR-R	GGGGACTGCTTTTTTGTACAAACTTGGCTTTGCTTGTTCT

Supplementary Table S3

Primers used for vector construction for production of transgenic plants

Name	Sequence (5′→3′)
ANAC046-F	GATGGTGGAAGAAGGCGGCGTAG
ANAC046-SRDX-R	GCTAGTATATAAATCTTCCCAGAAG
ANAC046-OX-R	TTAGCTAGTATATAAATCTTCCCAG

Supplementary Table S4

Primers used for quantitative real-time PCR

Name	Sequence (5′→3′)
ANAC046-RT-F	GGTTTCGGTTTCACCCTACTGATG
ANAC046-RT-R	ATGGCTCGTTCTTGTTGAGGTC
Actin-RT-F	TCCCGCTATGTATGTCGC
Actin-RT-R	ACCCTCGTAGATTGGCAC
